# High intact fibroblast growth factor 23 levels associated with low hemoglobin levels in patients on chronic hemodialysis

**DOI:** 10.3389/fmed.2023.1098871

**Published:** 2023-04-04

**Authors:** Yu-Wei Fang, Jing-Tong Wang, Tzu Yun Lin, Chung-Jen Lee, Tsrang-Neng Jang, Ming-Hsien Tsai, Hung-Hsiang Liou

**Affiliations:** ^1^Division of Nephrology, Department of Internal Medicine, Shin-Kong Wu Ho-Su Memorial Hospital, New Taipei City, Taiwan; ^2^Department of Medicine, School of Medicine, Fu Jen Catholic University, New Taipei City, Taiwan; ^3^Division of Nephrology, Department of Internal Medicine, Hsin-Jen Hospital, New Taipei City, Taiwan; ^4^Department of Nursing, Tzu Chi University of Science and Technology, Hualien, Taiwan; ^5^Department of Internal Medicine, Shin-Kong Wu Ho-Su Memorial Hospital, New Taipei City, Taiwan

**Keywords:** hemodialysis, intact fibroblast growth factor 23, C-terminal fibroblast growth factor 23, anemia, erythropoiesis

## Abstract

**Introduction:**

A negative association between C-terminal fibroblast growth factor 23 (cFGF23) and hemoglobin (Hb) levels has been reported in patients with predialysis chronic kidney disease. In dialysis patients, the dominant form of serum FGF23 is intact FGF23 (iFGF23); however, its association with the Hb level remains unclear. Therefore, simultaneously monitoring iFGF23 and cFGF23 levels is crucial. In this study, we investigated the associations between both forms of FGF23 (iFGF23 and cFGF23) and renal anemia in chronic hemodialysis (CHD) patients.

**Methods:**

We included 166 CHD patients from two hospitals in this cross-sectional, observational study. The primary predictors were serum iFGF23, cFGF23, and iFGF23/cFGF23 levels. The main outcome was the Hb level.

**Results:**

Among the CHD patients included, 60.8% were men with a mean age of 59.4 ± 12.7 years. In the crude analysis, iFGF23 and iFGF23/cFGF23 levels showed a significant negative association (−0.27, *p* = 0.004 and −0.22, *p* = 0.034, respectively) with the Hb level. Even after adjusting for multiple variables (a parsimonious model), every increment of natural log transformation by 1 for (ln)iFGF23 and ln(iFGF23/cFGF23) levels showed a negative correlation with the Hb level (estimate: −0.27 [95%CI: −0.44, −0.10, *p* = 0.001]; −0.19 [95%CI: −0.37, −0.01, *p* = 0.042], respectively), whereas both were positively associated with erythropoietin-stimulating agent (ESA) hyporesponsiveness (odds ratio [OR]: [95%CI: 2.30, 1.26–4.17], *p* = 0.006; 1.95 [95%CI: 1.08–3.50], *p* = 0.025). Moreover, these abovementioned associations were more dominant in patients with diabetes who used angiotensin receptor blockers.

**Discussion:**

In conclusion, a negative association between serum iFGF23 or iFGF23/cFGF23 level and the Hb level was observed in our CHD patients. Meanwhile, a higher iFGF23 or iFGF23/cFGF23 level may predispose patients to ESA hyporesponsiveness.

## Introduction

According to the data from the National Health and Nutrition Examination Survey, the prevalence of anemia can vary from 8.4 to 53.4% at different stages of chronic kidney disease (CKD) (1, 2). The causes of renal anemia are multifactorial and include decreased erythropoietin production, iron deficiency, malnutrition–inflammation status, 1,25-dihydroxyvitamin D (1,25(OH)_2_D) deficiency, and uremic toxin accumulation (3, 4). In clinical practice, renal anemia is usually accompanied by an impaired quality of life (5), a higher prevalence of cardiovascular disease (CVD) (6), congestive heart failure (7), and increased hospitalization and mortality rates (8).

The burgeoning link between CKD mineral bone disease and renal anemia has attracted attention in recent years. Fibroblast growth factor 23 (FGF23), an endocrine FGF subfamily protein, can increase urinary phosphate excretion and decrease intestinal phosphate absorption to counteract high phosphorus load in CKD patients (9, 10). Serum FGF23 can be found either in its biologically active form [intact FGF23 (iFGF23)] or its inactive fragment form [C-terminal FGF23 (cFGF23)].

Beyond its role in mineral homeostasis, FGF23 was demonstrated to impede prenatal and postnatal erythropoiesis in an animal experiment (11). In our previous study that focused on 53 incident stage 3–4 CKD patients, we first demonstrated that values higher than the median cFGF23 value were negatively correlated with hemoglobin (Hb) levels (12). Thereafter, several studies confirmed our findings either in mice or in non-dialysis CKD patients (13–18). In all of these studies, only cFGF23 was adopted. However, Shimada et al. demonstrated that the serum FGF23 in dialysis patients was mostly iFGF23 (19). This finding implies that increased production and impaired cleavage of FGF23 in CKD patients can lead to distorted proportions of active and inactive FGF23 (20). Impairments such as FGF23 cleavage and distorted FGF23 ratio have been reported to exaggerate along with the progression of CKD (19, 21). In fact, the iFGF23/cFGF23 ratio was higher in CKD patients than in the healthy population (20). Usui et al. reported that erythropoietin-stimulating agent (ESA) hyporesponsiveness was also associated with either the highest or lowest iFGF23 quintile in Japanese CHD patients (22).

Because the cleavage of FGF23 in chronic hemodialysis (CHD) alters the serum levels of iFGF23 and cFGF23 and the ratio of iFGF23/cFGF23, their relationship with serum Hb levels remains unclear. In this study, we examined the relationship between both forms of FGF23 (iFGF23 and cFGF23) and Hb levels in CHD patients.

## Materials and methods

### Patients

A total of 466 patients who had undergone HD for more than 3 months at Hsin-Jen Hospital and Shin-Kong Wu Ho-Su Memorial Hospital and were aged between ≥20 years and <85 years were screened for this cross-sectional, observational study. Patients with a poor nutritional status (body mass index [BMI] <17 kg/m^2^), kidney transplant history, acute hepatitis, autoimmune diseases, malignancy, myocardial infarction, or stroke within 6 months before enrollment, or hospitalization due to infection within 3 months before enrollment were excluded from the study. This study was performed as per the ethical principles of the Declaration of Helsinki and was approved by the ethics committee of Shin-Kong Wu Ho-Su Memorial Hospital (No. 20160802R). All patients gave their informed consent to participate in the study.

### Biochemical and clinical parameters

At the time of enrollment, the following data were recorded: age; gender; dialysis vintage; body mass index; history of diabetes (DM), hypertension, and coronary artery disease; and usage of angiotensin receptor blockers (ARBs), beta-blockers, calcium channel blockers, lipid-lowering agents, and ESA.

Fasting blood samples were collected and centrifuged at 3,000× *g* for 10 min, after which the supernatant serum was stored in a refrigerator at −20°C prior to analyses. An autoanalyzer (Beckman AU) was used for the analysis of parameters such as the white blood cell count (10^3^/uL), glucose (mg/dL), Hb (g/dL), blood urea nitrogen (mg/dL), creatinine (mg/dL), sodium (mEq/L), potassium (mEq/L), calcium (mg/dL), phosphate (mg/dL), alkaline phosphate (Alk-P, IU/L), albumin (g/dL), uric acid (mg/dL), total cholesterol (mg/dL), triglyceride (mg/dL), iron (μg/dL), ferritin (ng/mL), and iron saturation (iron/iron-binding capacity, %). High-sensitivity C-reactive protein levels (hs-CRP, mg/dL) were measured using the latex-enhanced immunoturbidimetric method (Simens ADVIA Chemistry Systems). Intact parathyroid hormone (iPTH, pg/mL) levels were measured using the Roche Elecsys assay (Roche Diagnostics, www.roche.com). FGF23 was measured using a two-site enzyme-linked immunosorbent assay that detects two epitopes in the carboxyl-terminal portion of FGF23 (cFGF23) and also detects epitopes within the amino-terminal and carboxyl-terminal portions of FGF-23 (iFGF23) (Quidel, www.quidel.com). 1,25(OH)_2_D [IDS, www.idspic.com], sclerostin (Biomedica, www.bmgrp.com), Dickkopf-1 (Biomedica, www.bmgrp.com), and α-Klotho (IBL America, www.ibl-america.com) levels were measured using a chemiluminescent immunoassay. The dialysis quality (Kt/V) was measured using the Daugirdas formula (23), and the cardiothoracic ratio was calculated by dividing the transverse diameter of the heart by the maximum inner width of the thoracic cavity using chest radiography.

The ESA prescribed for our CHD patients was either epoetin beta or darbepoetin alfa. However, the dose we administered was based on a protocol provided by the Bureau of National Health Insurance, according to which the ESA dose was escalated and had an upper limit. Thereafter, we defined the ESA dose in our study using the mean dosage prescribed 6 months before the recruitment. To standardize the ESA dose, we converted darbepoetin alfa to epoetin beta by adopting a ratio of 1 *ug*:200 IU. In our CHD patients, mean Hb levels of <10 g/dL and mean-standardized ESA doses of >6,000 u/week were regarded as ESA hyporesponsiveness, a definition we also adopted in other studies (22, 24).

### Statistical analyses

Clinical parameters are expressed as the mean ± standard deviation or median (25th, 75th centile; IQR) for continuous variables and frequencies with proportions for categorical variables. Pearson's correlation coefficient was used to examine correlations between variables with normally distributed data, whereas Spearman's rank correlation coefficient was used for variables whose data distributions deviated from the normal distribution. Wilcoxon signed-rank test or *t*-test was used to compare the means of continuous variables according to whether the data distributions were normal or not, and $X^{2}\;$ test was used to compare categorical variables between the groups. Some factors were naturally log-transformed (ln) to approximate a normal distribution.

A simple linear regression model was used to determine the risks associated with Hb levels. In the parsimonious model, the variables were chosen *via* the stepwise model selection method when *p* < 0.15 of the *F*-statistic for entry and *p* > 0.05 for removal. A separate regression analysis was used to model the changes in Hb levels as a function of ln(iFGF23) and ln(iFGF23/cFGF23) using a modified stepwise procedure with four modeling steps. In addition, logistic regression was used to model ESA hyporesponsiveness as a function of ln(iFGF23) and ln(iFGF23/cFGF23). A two-sided *p*-value of <0.05 was considered statistically significant. All statistical analyses were performed using SAS for Windows version 9.4 (SAS Institute Inc., Cary, NC, USA).

## Results

### Patient characteristics

**Figure 1:**
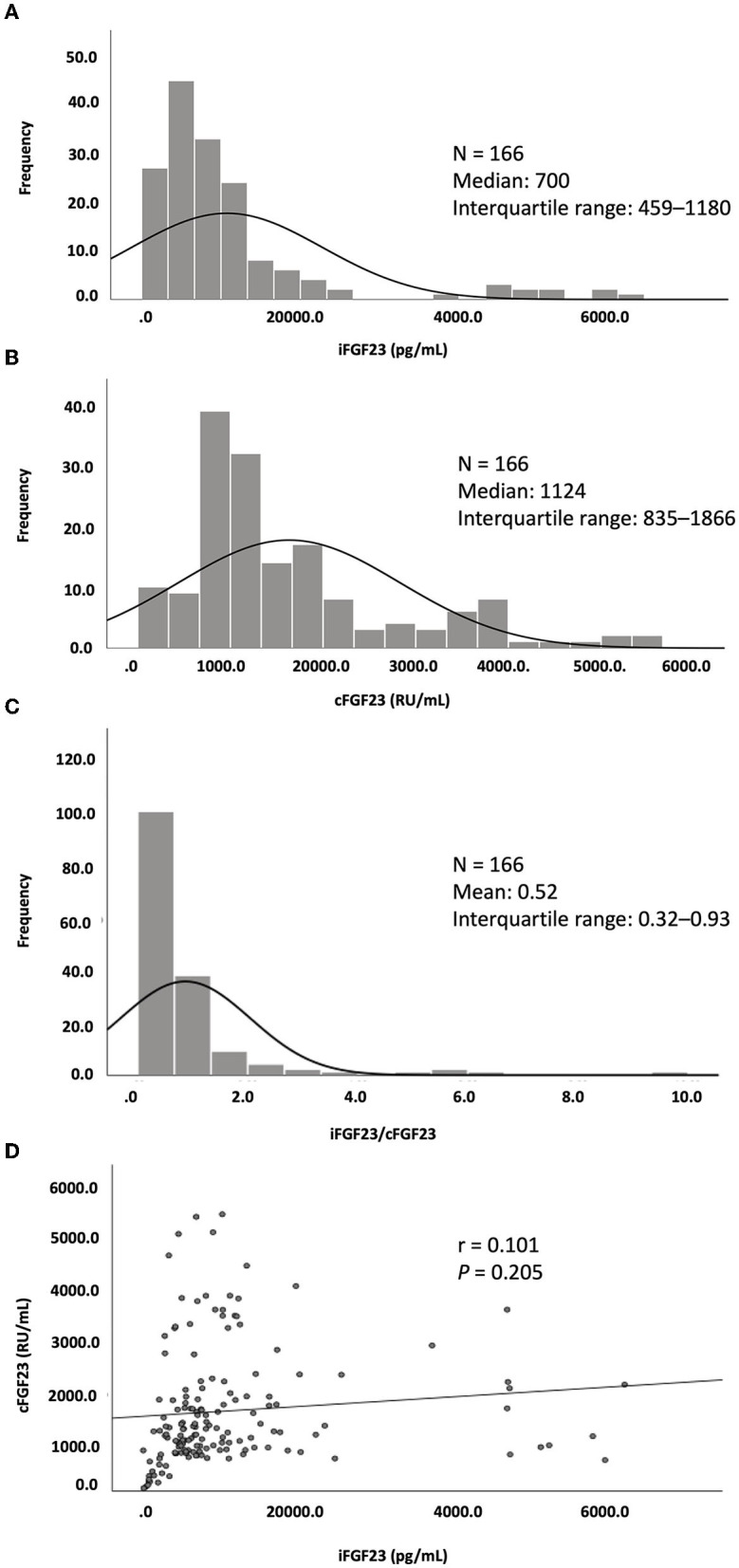
Distribution of **(A)** iFGF23, **(B)** cFGF23, and **(C)** iFGF23/cFGF23 and **(D)** the correlation between iFGF23 and cFGF23.

**Figure 2:**
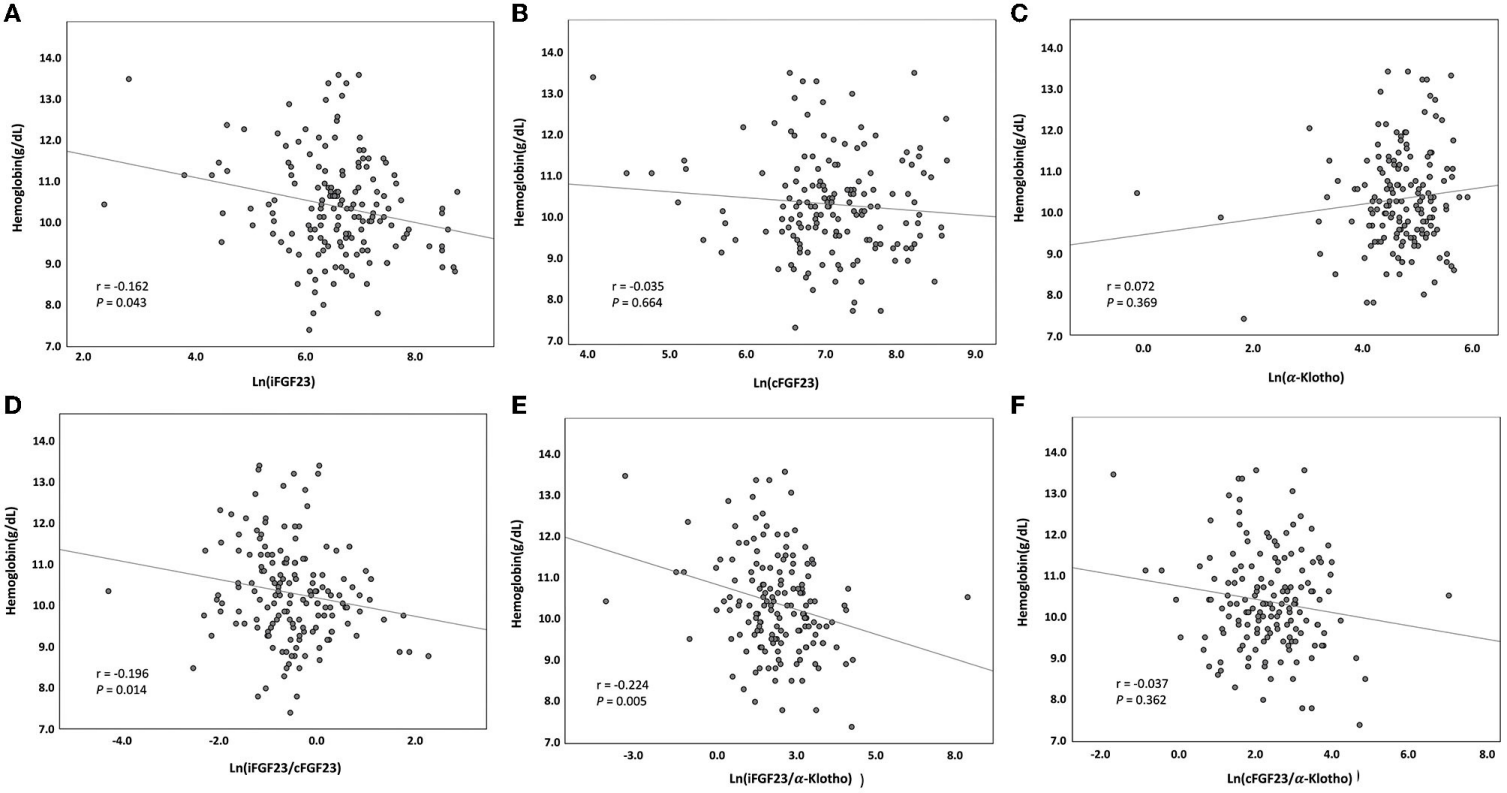
Serum hemoglobin level vs. serum **(A)** ln(iFGF23), **(B)** ln(cFGF23), **(C)** ln(α-klotho), **(D)** ln(iFGF23/cFGF23), **(E)** ln(iFGF23/α-klotho), and **(F)** ln(cFGF23/α-klotho) levels. The lines indicate best-fit regression lines derived using the least mean square method.

Among the CHD patients screened, only 166 patients with a mean age of 59.4 ± 12.7 years were recruited. The median dialysis vintage of these patients was 6.1 years [interquartile range (IQR): 2.8–11.7; 61% (*n* = 101) were men, 49% (*n* = 81) were diabetic, and 55% (*n* = 91) had a history of coronary artery disease]. With a mean Hb level of 10.3 ± 1.2 g/dL, 40.3% (*n* = 67) of the patients had Hb levels of <10 g/dL (Table 1). A high missing rate (15.6%) of the hs-CRP data was noted. Serum iFGF23, cFGF23, and iFGF23/cFGF23 levels were measured, and the median levels were 700 pg/mL (IQR: 459–1,180) for iFGF23, 1124 RU/mL (IQR: 835–1,966) for cFGF23, and 0.52 (IQR: 0.32–0.93) for iFGF23/cFGF23. In addition, the distributions of iFGF23, cFGF23, and iFGF23/cFGF23 were all right-skewed (Figures 1A–C), and there was no significant correlation between iFGF23 and cFGF23 (Figure 1D). Moreover, Hb levels were negatively associated with ln(iFGF23), ln(iFGF23/cFGF23), or ln(iFGF23/α-klotho). These findings are illustrated in Figure 2.

**Table 1:** Characteristics of the study population.

**Characteristics**	**All (*n* = 166)**	**Hb <10 g/mL (*n =* 67)**	**Hb ≥10 g/mL (*n =* 99)**	***p* value**
**Basic information**				
Age (years)	59.4 ± 12.7	60.9 ± 10.9	58.3 ± 13.7	0.179
Males [*n* (%)]	101 (60.8)	39 (58.2)	62 (62.3)	0.567
Duration of HD (year)	6.1 (2.8, 11.7)	5.1 (2.4, 10.2)	6.7 (3.2, 12.1)	0.246
Body mass index (kg/m^2^)	23.6 ± 4.0	23.9 ± 4.0	23.3 ± 4.0	0.307
Cardiothoracic ratio (%)	47.9 ± 5	48.2 ± 4.9	47.9 ± 4.8	0.559
**Comorbidities**				
Diabetes mellitus [*n* (%)]	81 (48.8)	39 (58.2)	42 (42.4)	0.045
Hypertension [*n* (%)]	109 (65.7)	43 (64.2)	66 (66.7)	0.740
CAD [*n* (%)]	91 (54.8)	40 (59.7)	51 (51.5)	0.298
**Bone-related markers**				
iFGF23 (pg/mL)	700 (459, 1,180)	746 (459, 1,443)	685 (432, 1,086)	0.157
cFGF23 (RU/mL)	1,124 (835, 1,966)	1,326 (834, 2,375)	1,219 (836, 1,868)	0.646
α-Klotho (pg/mL)	103 (77, 160)	104 (73, 154)	103 (78, 165)	0.849
iFGF23/cFGF23 ratio	0.52 (0.32 ,0.93)	0.56 (0.37, 1.07)	0.48 (0.29, 0.89)	0.134
iFGF23/α-Klotho ratio	7.0 (3.7, 15.1)	7.7 (4.2, 21.4)	6.0 (3.6, 11.7)	0.020
cFGF23/α-Klotho ratio	12.6 (6.3, 24.2)	13.2 (6.4, 26.2)	12.2 (6.2, 22.8)	0.363
DKK1 (pmol/L)	3.37 (1.67, 5.14)	2.39 (1.21, 5.09)	3.66 (2.29, 5.28)	0.053
Sclerostin (pmol/L)	146 (111, 198)	158 (107, 217)	143 (112, 192)	0.364
1,25(OH)_2_D (pmol/L)	7.04 (2.98, 17.13)	7.07 (3.1, 19.6)	6.7 (2.9, 16.7)	0.997
iPTH (pg/mL)	213 (89, 384)	194 (76, 344)	215 (98, 423)	0.283
**Other dialysis biochemistry**				
WBC count (10^3^/ul)	6.6 ± 2.1	6.8 ± 2.8	6.5 ± 1.6	0.466
Hemoglobin (g/dL)	10.3 ± 1.2	9.2 ± 0.6	11.1 ± 0.9	<0.001
BUN (mg/dL)	74 ± 17	72.3± 19.4	75.8 ± 15.3	0.225
Creatinine (mg/dL)	9.7 ± 2.0	9.2 ± 1.8	10.1 ± 2.1	0.011
Sodium (meq/L)	137 ± 3	136.9 ± 3.1	137.5 ± 2.8	0.217
Potassium (meq/L)	4.7 ± 0.6	4.6 ± 0.6	4.8 ± 0.6	0.022
Calcium (mg/dL)	9.5 ± 1.0	9.3 ± 0.8	9.6 ± 1.0	0.165
Phosphate (mg/dL)	4.9 ± 1.2	4.7 ± 1.3	5.0 ± 1.0	0.064
Alk-P (U/L)	70 (52, 98)	65 (50, 92)	71 (52, 101)	0.587
Albumin (g/dL)	4.1 ± 0.3	4.0 ± 0.3	4.1 ± 0.3	0.135
Uric acid (mg/dL)	6.3 ± 1.3	6.4 ± 1.4	6.2 ± 1.2	0.384
Total Cholesterol (mg/dL)	161 ± 33	160.4 ± 35.0	161.9 ± 32.3	0.779
Triglyceride (mg/dL)	116 (76,185)	128 (85, 216)	111 (71, 181)	0.123
Iron (μg/dL)	74 ± 31	72.0 ± 38.8	76.0 ± 25.3	0.464
Ferritin (ng/mL)	437 (243, 583)	549 (453, 669)	432 (209, 560)	0.072
TSAT (%)	29.8 ± 12.6	28.5 ± 14.3	30.6 ± 11.4	0.319
hs-CRP^*^ (mg/dL)	0.21 (0.06, 0.64)	0.44 (0.10, 1.07)	0.17 (0.05, 0.41)	0.005
Kt/V	1.4 ± 0.2	1.4 ± 0.2	1.4 ± 0.2	0.750
**Medications**				
ARBs [*n* (%)]	65 (39.2)	31 (46.3)	34 (34.3)	0.122
Beta-blocker [*n* (%)]	76 (45.8)	29 (43.2)	47 (47.5)	0.594
CCB [*n* (%)]	78 (45.8)	34 (50.8)	44 (44.4)	0.424
Lipid-lowering agents [*n* (%)]	72 (43.4)	29 (43.3)	43 (43.4)	0.984
ESA (U/week)^#^	4,667 (3,500, 5,333)	5,667 (5,000, 6,667)	4,000 (2,833, 5,000)	<0.001

Values are expressed as the number (%) of patients, mean ± standard deviation, or median (interquartile range). Hb, hemoglobin; HD, hemodialysis; CAD, coronary artery disease; iFGF23, intact fibroblast growth factor 23; cFGF23, c-terminal fibroblast growth factor 23; DKK1, dickkopf-1; 1,25(OH)2D, 1,25-(OH)2 vitamin D; iPTH, intact parathyroid hormone; WBC, white blood cells; BUN, blood urea nitrogen; Alk-P, alkaline phosphatase; TSAT, transferrin saturation; hs-CRP, high sensitive C-reactive protein; Kt/V, urea kinetics; ARBs, angiotensin-receptor blockers; CCB, calcium channel blockers; ESA, erythropoietin-stimulating agents.

* A missing rate of 15.6%.

# The mean dosage 6 months prior to enrollment.

### Determinants of serum Hb levels in CHD patients

When stratified by Hb level, in the population of patients with Hb <10 g/dL, there were significantly more diabetic people (58.2% [*n* = 39] vs. 42.4% [*n* = 42], *p* = 0.045), higher iFGF23/α-klotho ratios [7.7 (IQR: 4.2–21.4) vs. 6.0 (IQR: 3.6–11.7), *p* = 0.020], higher hs-CRP levels [0.44 mg/dL (IQR: 0.1–1.07) vs. 0.17 (IQR: 0.05–0.41), *p* = 0.005], higher mean-standardized ESA doses [5,667 u/wk (IQR: 5,000–6,667) vs. 4,000 u/wk (IQR: 2,833–5,000), *p* < 0.001], lower serum Cr levels (9.2 ± 1.8 mg/dL vs. 10.1 ± 2.1 mg/dL, *p* = 0.011), and lower serum K levels (4.6 ± 0.6 mg/dL vs. 4.8 ± 0.6 mg/dL, *p* = 0.022). No significant differences in serum albumin, iFGF23, cFGF23, Alk-P, iPTH, 1,25(OH)_2_D, iron, ferritin, or transferrin saturation were observed (Table 1).

When we applied linear regression to analyze the risk factors for Hb level, the crude analysis results revealed that diabetes [−0.38; 95% confidence interval (CI): −0.74, −0.01, *p* = 0.043], ln(iFGF23) (−0.27; 95%CI: −0.45, −0.84, *p* = 0.004), ln(iFGF23/cFGF23) (−0.22; 95% CI: −0.43, −0.02, *p* = 0.034), ln(iFGF23/α-klotho) (−0.24; 95% CI: −0.39, −0.09, *p* = 0.001), creatinine (0.11; 95% CI: 0.02, 0.20, *p* = 0.019), sodium (0.06; 95% CI: <0.001, 0.13 *p* = 0.048), albumin (0.87; 95%CI: 0.28, 1.46, *p* = 0.004), ln(alk-P) (0.46; 95% CI: 0.09, 0.83 *p* = 0.014), ln(ferritin) (−0.32; 95% CI: −0.49, −0.15, *p* < 0.001), and the use of ARBs (−0.40; 95% CI: −0.77, −0.02, *p* = 0.037) were significant determinants in our CHD patient cohort.

After multivariable adjustment using the forward stepwise model selection method, among the 39 parameters listed in Table 2, only eight parameters were identified as the main determinants (a parsimonious model). These parameters were age (0.15; 95% CI: 0.01, 0.29 every 10 years), ln(iFGF23) (−0.27; 95% CI: −0.44, −0.10), sodium (0.07; 95% CI: 0.02, 0.13), phosphate (0.15; 95% CI: 0.01, 0.31), albumin (0.88; 95% CI: 0.30, 1.46), Alk-P (0.63; 95% CI: 0.30, 0.95), ln(ferritin) (−0.42; 95% CI: −0.58, −0.26), and use of ARBs (−0.49; 95% CI: −0.82, −0.16).

**Table 2:** The risk analysis for the serum hemoglobin level in patients with chronic hemodialysis.

**Parameters**	**Crude**		**Multivariable**	
	**Estimate (95% CI)**	***p*** **value**	**Estimate (95% CI)**	***p*** **value**
**Basic information**				
Age (per 10 years)	0.001 (−0.15, 0.15)	0.984	0.15 (0.01, 0.29)	0.032
Males [vs. female]	0.33 (−0.05, 0.70)	0.089		
Duration of HD (per 10 year)	0.06 (−0.21, 0.33)	0.664		
Body mass index (per 1 kg/m^2^)	−0.02 (−0.06, 0.03)	0.482		
Cardiothoracic ratio (per 1 %)	−0.21 (−4.06, 3.63)	0.913		
**Comorbidities**				
Diabetes mellitus [vs. none]	−0.38 (−0.74, −0.01)	0.043		
Hypertension [ vs. none]	−0.10 (−0.49, 0.29)	0.601		
CAD [ vs. none]	−0.07 (−0.44, 0.31)	0.722		
**Bone–related markers**				
Ln(iFGF23) (per 1 unit)	−0.27 (−0.45, −0.84)	0.004	−0.27 (−0.44, −0.10)	0.001
Ln(cFGF23) (per 1 unit)	−0.14 (−0.38, 0.10)	0.244		
Ln(α-Klotho) (per 1 unit)	0.19(−0.07, 0.44)	0.143		
Ln(iFGF23/cFGF23) (per 1 unit)	−0.22 (−0.43, −0.02)	0.034		
Ln(cFGF23/α-Klotho) (per 1 unit)	−0.16 (−0.33, 0.01)	0.068		
Ln(iFGF23/α-Klotho) (per 1 unit)	−0.24 (−0.39, −0.09)	0.001		
Ln(DKK1) (per 1 unit)	0.17(−0.04, 0.38)	0.120		
Ln(Sclerostin) (per 1 unit)	−0.05 (−0.32, 0.22)	0.712		
Ln(1,25(OH)_2_D) (per 1 unit)	−0.01 (−0.13, 0.10)	0.839		
Ln(iPTH) (per 1unit)	0.09 (−0.06, 0.24)	0.229		
**Other dialysis biochemistry**				
WBC count (per 1 10^3^/ul)	−0.24 (−0.39, −0.09)	0.001		
Blood nitrogen (per 1 mg/dL)	0.002 (−0.008, 0.013)	0.656		
Creatinine ( per 1 mg/dL)	0.11 (0.02, 0.20)	0.019		
Sodium (per 1 mg/dL)	0.06 (0.00, 0.13)	0.048	0.07 (0.02, 0.13)	0.012
Potassium (per 1 mEq/L)	0.33 (0.03, 0.63)	0.028		
Calcium (per 1 mg/dL)	0.15 (−0.04, 0.34)	0.110		
Phosphate (per 1 mg/dL)	0.12 (−0.04, 0.28)	0.150	0.15 (0.01, 0.31)	0.042
Ln(Alk-P) ( per 1 unit)	0.46 (0.09, 0.83)	0.014	0.63 (0.30, 0.95)	<0.001
Albumin (per 1 g/dL)	0.87 (0.28, 1.46)	0.004	0.88 (0.30, 1.46)	0.003
Uric acid (per 1 mg/dL)	0.10 (−0.24, 0.04)	0.159		
Total cholesterol (per 1 mg/dL)	0.00 (−0.01, 0.01)	0.997		
Ln(Triglyceride) (per 1 unit)	−0.18 (−0.47, 0.12)	0.244		
Iron (μg/dL) (per 1 unit)	0.003 (−0.003, 0.009)	0.315		
Ln(Ferritin) (per 1 unit)	−0.32 (−0.49, −0.15)	<0.001	−0.42 (−0.58, −0.26)	<0.001
TSAT (per 1 %)	0.00 (−0.01, 0.02)	0.651		
Hs-CRP (per 1 mg/dL)	−0.02 (−0.25, 0.21)	0.885		
Kt/V (per 1 unit)	−0.39 (−0.40, 0.62)	0.449		
**Medications**				
ARBs [vs. none]	−0.40 (−0.77, −0.02)	0.037	−0.49 (−0.82, −0.16)	0.003
Beta-blocker [vs. none]	−0.21 (−0.58, 0.17)	0.275		
CCB [vs. none]	−0.25 (−0.62, 0.12)	0.186		
Lipid-lowering agents [vs. none]	−0.14 (−0.51, 0.24)	0.476		

HD, hemodialysis; CAD, coronary artery disease; Ln, natural log-transformation; iFGF23, intact fibroblast growth factor 23; cFGF23, c-terminal fibroblast growth factor 23; DKK1, dickkopf-1; 1,25(OH)2D, 1,25-(OH)2 vitamin D; iPTH, intact parathyroid hormone; WBC, white blood cells; BUN, blood urea nitrogen; Alk-P, alkaline phosphatase; TSAT, transferrin saturation; hs-CRP, high sensitive C-reactive protein; Kt/V, urea kinetics; ARBs, angiotensin-receptor blockers; CCB, calcium channel blockers.

### Association between FGF23 and Hb levels in CHD patients

After adjusting for demographic data, comorbidities, dialysis-related parameters, and bone markers using stepwise multivariable adjusting models, increments of not only ln(iFGF23) (−0.27, 95% CI, −0.44, −0.10) but also ln(iFGF23/cFGF23) (−0.19, 95% CI, −0.37, −0.01) were negatively associated with Hb levels, and these associations remained significant in crude analyses and all adjustment models (Table 3).

**Table 3:** Association between FGF23 and serum hemoglobin level in patients on chronic hemodialysis.

**Model**	**Every 1 increment of ln(iFGF23)**		**Every 1 increment of ln(iFGF23/cFGF23)**	
	**Estimate (95% CI)**	***P*** **value**	**Estimate (95% CI)**	***P*** **value**
Crude	−0.27 (−0.45, −0.08)	0.004	−0.22 (−0.43, −0.02)	0.034
Model 1	−0.33 (−0.52, −0.14)	<0.001	−0.30 (−0.51, −0.08)	0.006
Model 2	−0.32 (−0.51, −0.13)	0.001	−0.31 (−0.51, −0.10)	0.004
Model 3	−0.27 (−0.44, −0.10)	0.001	−0.19 (−0.37, −0.01)	0.042

Multivariate model 1 is adjusted for age, sex, hemodialysis vintage, body mass index, cardiothoracic ratio, diabetes mellitus, hypertension, and coronary artery disease. Multivariate model 2 comprises model 1 as well as adjustments for ln(α-Klotho), ln (Dickkopf-1), ln(sclerostin), ln(1,25-(OH)2 vitamin D), ln(intact parathyroid hormone), white blood cells count, blood nitrogen, creatinine, sodium, potassium, calcium, phosphate, ln(alkaline phosphatase), albumin, uric acid, total cholesterol, ln(triglyceride), ln(ferritin), transferrin saturation, urea kinetics, angiotensin receptor blockers, beta-blocker, calcium channel blockers, and lipid-lowering agents. Multivariate model 3 (a parsimonious model) comprises age, sodium, phosphate, albumin, ln(alkaline phosphatase), ln(ferritin), and angiotensin receptor blockers. Ln, natural log transformation; CI, confidence interval; iFGF23, intact fibroblast growth factor 23; cFGF23, C-terminal fibroblast growth factor 23.

### Subgroup analysis for the association between FGF23 and Hb levels

**Figure 3:**
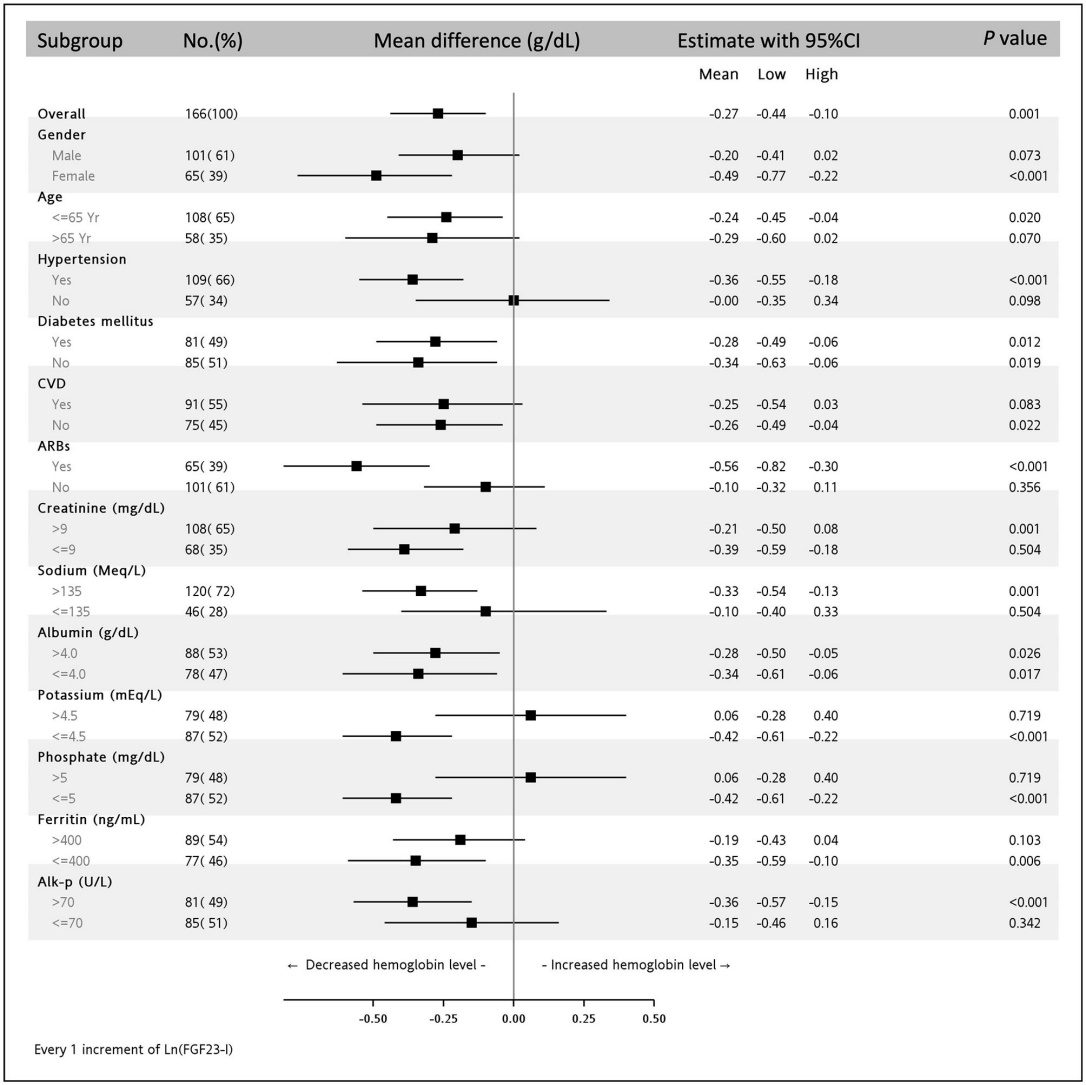
Subgroup analysis of the association between ln(iFGF23) and serum hemoglobin level in the multivariable adjusting model. The parsimonious adjusting model is Model 3 in Table 3.

As shown in Figure 3, after multivariable adjustments, discrete associations between high ln(iFGF23) and low Hb levels were noted in women aged ≤65 years who had a history of hypertension, CVD, or ARB usage and whose serum Cr level was ≤9 mg/dL, sodium >135 mEq/L, potassium ≤4.5 mEq/L, phosphate ≤5 mEq/L, ferritin ≤400 ng/mL, or alk-P >70 U/L (all *p*-values <0.05).

A further subgroup analysis of DM and ARBs use is shown in Table 4. It shows that only the subgroup of CHD patients with DM and ARB use retained a significant negative association between ln(iFGF23) or ln(iFGF23/cFGF23) and H, in which the estimates of ln(iFGF23) and ln(iFGF23/cFGF23) were −0.45 (−0.78, −0.12) and −0.29 (−0.58, −0.01), respectively.

**Table 4:** The association of FGF23 and hemoglobin in the groups of DM and ARBs.

**Groups**	**N**	**Every 1 increment of ln(iFGF23)**		**Every 1 increment of ln(iFGF23/cFGF23)**	
		**Estimate (95% CI)**	***p*** **value**	**Estimate (95% CI)**	***p*** **value**
DM (+) and ARBs (+)	37	−0.45 (−0.78, −0.12)	0.008	−0.29 (−0.58, −0.01)	0.045
DM (+) and ARBs (—)	44	−0.15 (−0.46, 0.16)	0.334	0.04 (−0.23, 0.41)	0.838
DM (–) and ARBs (+)	28	−0.55 (−1.14, 0.04)	0.064	−0.46 (−1.05, 0.13)	0.118
DM (–) and ARBs (–)	57	−0.11 (−0.45, 0.23)	0.516	0.17 (−0.27, 0.60)	0.446

The multivariable adjusting model comprises age, sodium, phosphate, albumin, ln(alkaline phosphatase), and ln(ferritin). Ln, natural log transformation; CI, confidence interval; iFGF23, intact fibroblast growth factor 23; cFGF23, c-terminal fibroblast growth factor 23; DM, diabetes mellitus; ARBs, angiotensin-receptor blockers.

### Association between ESA hyporesponsiveness and FGF23 in CHD patients

Table 5 shows that 13.9% of enrolled patients (23/166) had ESA hyporesponsiveness. Moreover, in the stepwise regression models, both ln(iFGF23) and ln(iFGF23/cFGF23) were positively associated with ESA hyporesponsiveness in the parsimonious model (odds ratio [OR]: 2.30, 95%CI, 1.26–4.17; 1.95, 95%CI, 1.08–3.50, respectively).

**Table 5:** Association between FGF23 and ESA hyporesponsiveness in patients on chronic hemodialysis.

**Model**	**ESA hyporesponsiveness (yes vs. no)^#^** **(ESA** > **6,000 u/week and hemoglobin**<**10 g/dL)**			
	**Every 1 increment of ln(iFGF23)**		**Every 1 increment of ln(iFGF23/cFGF23)**	
	**Odds ratio (95% CI)**	***p*** **value**	**Odds ratio (95% CI)**	***p*** **value**
Crude	1.68 (1.02–2.77)	0.042	1.46 (0.90–2.37)	0.121
Model 1	2.21 (1.16–4.23)	0.016	1.90 (1.02–3.54)	0.043
Model 2	3.52 (1.27–9.78)	0.015	2.26 (0.92–5.55)	0.074
Model 3	2.30 (1.26–4.17)	0.006	1.95 (1.08–3.50)	0.025

Multivariate model 1 is adjusted for age, sex, hemodialysis vintage, body mass index, cardiothoracic ratio, diabetes mellitus, hypertension, and coronary artery disease. Multivariate model 2 comprises model 1 as well as adjustments for ln(α-Klotho), ln (Dickkopf-1), ln(sclerostin), ln(1,25-(OH)2 vitamin D), ln(intact parathyroid hormone), white blood cells count, blood nitrogen, creatinine, sodium, potassium, calcium, phosphate, ln(alkaline phosphatase), albumin, uric acid, total cholesterol, ln(triglyceride), ln(ferritin), transferrin saturation, urea kinetics, angiotensin receptor blockers, beta-blockers, calcium channel blockers, and lipid-lowering agents. Multivariate model 3 (a parsimonious model) comprises creatinine, uric acid, and ln(triglyceride). iFGF23, intact fibroblast growth factor 23; cFGF23, C-terminal fibroblast growth factor 23; ESA, erythropoiesis-stimulating agents; ln, natural log transformation; CI, confidence interval.

# There were 23 participants who had ESA hyporesposiveness.

## Discussion

In this study, negative associations of serum iFGF23 level and iFGF23/cFGF23 ratio with Hb levels were found in CHD patients. Furthermore, serum iFGF23 level and iFGF23/cFGF23 ratios were positively associated with ESA hyporesponsiveness. These findings were observed in patients with predialysis CKD as well as those who had undergone HD. According to our subgroup analysis, women aged ≤65 years who used ARBs and had a higher serum iFGF23 level or a lower serum ferritin level were independently correlated with lower Hb levels. This negative association between serum iFGF23 or iFGF23/cFGF23 and Hb levels was more dominant in our CHD patients who were diabetics and used ARBs.

Iron deficiency has been recognized as one of the causes of anemia (25). In fact, iron deficiency is associated with higher FGF23 levels in women with a history of heavy uterine bleeding and in patients undergoing HD (26, 27). In HD patients, high FGF23 levels can be lowered by supplementing intravenous iron or iron-containing phosphate binders (27–29). However, after adjusting for the iron status of our CHD patients, iFGF23 levels and iFGF23/cFGF23 ratios were still negatively correlated with Hb levels. In addition to iron, FGF23 itself has been demonstrated to induce anemia by intervening in erythropoiesis (11, 14, 30, 31). Coe et al. reported that both erythroid progenitor cell counts in the bone marrow and serum erythropoietin levels were higher in mice in which FGF23 was genetically inactivated compared with wide-type mice (11). Conversely, high FGF23 and low Hb levels, which were noted in five out of six nephrectomized mice, could be rescued by applying an FGF23-blocking peptide (14). Several studies have demonstrated an inverse correlation between FGF23 and erythropoiesis in clinical settings (29, 32–35). Recently, Usui et al. reported that Japanese CHD patients who had either the highest or the lowest iFGF23 quintile showed hyporesponsiveness to ESA (22). In their study, CHD patients in the lowest quintile had a poorer nutritional status than other participants. This could be the reason why we observed ESA hyporesponsiveness only in patients with higher FGF23 levels in our cohort since malnourished patients were excluded from our study.

Inflammation status in CHD patients has been shown to be another risk factor for anemia (25). Moreover, chronic inflammation in CKD patients has been reported to enhance the overproduction of iFGF23 (36). However, after adjusting for inflammatory markers, iFGF23 levels and iFGF23/cFGF23 ratios were all negatively correlated with Hb levels in our CHD patients.

Alon et al. demonstrated that 1,25(OH)_2_D could enhance the proliferative response of erythroid-origin stem cells to erythropoietin as well as increase erythropoietin receptor expression (37). In clinical practice, 1,25(OH)_2_D deficiency is regarded as an independent risk factor for renal anemia in CKD (38). However, after adjusting for serum 1,25(OH)_2_D in our CHD patients whose serum 1,25(OH)_2_D levels were relatively lower, iFGF23 or iFGF23/cFGF23 and Hb levels remained negatively correlated.

Inhibition of the RAA system, which could increase the renal plasma flow, led to reduced erythropoietin production in spontaneously hypertensive rats (39). Angiotensin II, through activation of the angiotensin II type 1 receptor, can also induce erythroid progenitor cell proliferation, whereas ARBs can antagonize such effects (40). In a retrospective study, type 2 diabetic CKD patients who were treated with ARBs had lower Hb levels (41). Our data also confirmed this finding (but in CHD patients). Moreover, hyporesponsiveness to erythropoietin-stimulating agents was demonstrated in chronic dialysis patients who used ARBs (42). According to our subgroup analysis, the negative correlation between iFGF23 and iFGf23/cFGF23 and renal anemia is presented only in diabetic CHD patients who used ARBs. Nevertheless, the mechanism underlying this finding is yet to be explored (43).

Blood-circulating FGF23 can be found either in its biologically active intact form or as inactive C-terminal fragments (19, 44). Owing to the heterogeneity of study designs caused due to the adoption of different FGF23 assays, the interpretation of FGF23 levels in clinical settings is controversial (19). In predialysis CKD patients, a negative correlation was delineated only between serum cFGF23 and Hb levels (12, 13, 15). However, the impaired cleavage of FGF23 in CKD patients makes serum iFGF23 and cFGF23 levels and the iFGF23/cFGF23 ratio distinct from those in normal subjects (19, 21, 22). In fact, the major form of serum FGF23 in peritoneal dialysis patients was found to be iFGF23 by Shimada et al. (19). Moreover, the iFGF23/cFGF23 ratio in CKD patients was found to be higher than that in the normal population (20). During the dialysis sessions, the smaller molecular weight of cFGF23 rendered it more dialyzable than iFGF23. In addition, iFGF23 remained biologically active even after dialysis (19). In the J-DOPPS cohort, Usui et al. demonstrated that iFGF23 had a negative impact on ESA resistance in CHD patients, although they did not assess cFGF23 in their study (30). In the present study, we analyzed iFGF23 and cFGF23 levels and the iFGF23/cFGF23 ratio and found that only the iFGF23 level and the iFGF23/cFGF23 ratio were correlated with the severity of anemia in CHD patients. Meanwhile, we also detected that this significant association of the iFGF23/cFGF23 ratio with the Hb level was mostly affected by the iFGF23 level.

In our CHD patients, the correlations between ln(iFGF23) and ln(iFGF23/cFGF23) and Hb were statistically significant but not so powerful (r = −0.162, *p* = 0.043; r = −0.196, *p* = 0.014). In addition, according to the subgroup analysis, this association was more predominant in diabetic CHD patients who used ARBs. These findings indicate that beyond FGF23, there could be some coexisting factors that are involved in renal anemia, and these factors are to be explored in greater detail.

The present study has several limitations. First, it was a cross-sectional study; thus, a causal relationship between iFGF23 and anemia in CHD patients could not be inferred. Second, the number of CHD patients included in this study was relatively small. Nevertheless, a significant correlation between the iFGF23 level and iFGF23/cFGF23 ratio and Hb level was observed. To avoid over-adjusting, we applied a stepwise model selection method to reduce the dimensionality in the multivariable model. Third, even if we avoided administering excessive and limited doses of ESAs by administering the mean-standardized ESA dose, it is still difficult to exclude the bias that may have been introduced. However, we still found that iFGF23 exerted a negative impact on ESA hyporesponsiveness in our CHD patients. Finally, although the patients were enrolled from two different hospitals, our data are not representative of CHD patients nationwide. Therefore, to verify our findings, multiple, large-scale multicenter studies need to be conducted. Finally, no healthy subjects or predialysis CKD patients were included as a control group in this study.

## Conclusion

In the present study, we extend the role of FGF23 in anemia from predialysis CKD patients to CHD patients. Instead of cFGF23 in predialysis CKD patients, the iFGF23 level and the iFGF23/cFGF23 ratio were negatively associated with the Hb levels of CHD patients. Further studies are required to confirm our findings.

## Data availability statement

The raw data supporting the conclusions of this article will be made available by the authors, without undue reservation.

## Ethics statement

The studies involving human participants were reviewed and approved by Shin-Kong Wu Ho-Su Memorial Hospital Ethics Committee (No. 20160802R). The patients/participants provided their written informed consent to participate in this study.

## Author contributions

Y-WF, M-HT, and H-HL: conceptualization. C-JL and M-HT: formal analysis. Y-WF: funding acquisition. Y-WF, J-TW, and TL: investigation. M-HT and H-HL: methodology and writing—reviewing and editing. J-TW, TL, and T-NJ: project administration. T-NJ, Y-WF, M-HT, and H-HL: resources. C-JL: validation. Y-WF and M-HT: writing—original draft. All authors contributed equally to this study and approved the manuscript.
